# Endosymbiosis in trypanosomatids: the bacterium regulates the intermediate and oxidative metabolism of the host cell

**DOI:** 10.1128/msphere.00457-25

**Published:** 2025-10-13

**Authors:** Azuil Barrinha, Ana Carolina Loyola-Machado, Marlon Dias Mariano dos Santos, Paulo Costa Carvalho, Wanderley de Souza, Ana Paula Valente, Antonio Galina, Maria Cristina Machado Motta

**Affiliations:** 1 Laboratório de Ultraestrutura Celular Hertha Meyer, Centro de Pesquisa em Medicina de Precisão (CPMP), Instituto de Biofísica Carlos Chagas Filho, Universidade Federal do Rio de Janeiro-UFRJ 204227 , Rio de Janeiro, Rio de Janeiro, Brazil; 2 Laboratory for Structural and Computational Proteomics, Carlos Chagas Institute, Fiocruz, Paraná, Brazil; 3 Instituto Nacional de Ciência e Tecnologia em Biologia Estrutural e Bioimagens – Rio de Janeiro 206381 , Rio de Janeiro, Brazil; 4 Centro Nacional de RMN, Departamento de Biologia Estrutural, Universidade Federal do Rio de Janeiro 28125, Rio de Janeiro, Brazil; 5 Laboratório de Bioenergética e Fisiologia Mitocondrial, Instituto de Bioquímica Médica Leopoldo de Meis, Universidade Federal do Rio de Janeiro 28125, Rio de Janeiro, Brazil; University at Buffalo - Downtown Campus, Buffalo, New York, USA

**Keywords:** symbiosis, trypanosomatids, intermediate metabolism, oxidative phosphorylation, fermentation, cell evolution

## Abstract

Endosymbiosis in trypanosomatids involves a mutualistic association between a symbiotic bacterium and a host protozoan and represents an excellent model for studying metabolic coevolution and the origin of organelles. This work investigated the influence of the symbiont on the metabolism of *Angomonas deanei* by comparing wild-type and aposymbiotic strains under different nutritional conditions. The presence of the symbiont enhanced cell proliferation in the medium containing a single carbon source and increased O₂ consumption. Wild-type cells utilized oxidative phosphorylation to produce ATP, whereas aposymbiotic cells relied on substrate-level glycolysis, resulting in the excretion of greater amounts of fermentative products, such as acetate, succinate, and ethanol. Proteomic analysis revealed an increased expression of glycolytic and fermentative enzymes by the aposymbiotic strain and oxidative phosphorylation enzymes by symbiont-harboring cells. These findings highlight the role of the symbiotic bacterium in optimizing host metabolism and provide insights into the evolution of parasitism in trypanosomatids when *A. deanei* is compared with pathogenic species.

**IMPORTANCE:**

This work provides groundbreaking insights into the metabolic and evolutionary dynamics of endosymbiosis, a topic of central importance to cellular evolution. Angomonas deanei, a trypanosomatid species, has become a paradigm for investigating the evolution of eukaryotic cells and the origin of organelles through endosymbiosis. Harbored in the cytoplasm of this protozoan, the symbiont engages in intricate metabolic exchanges, offering a time window to analyze the processes and evolutionary history that underlie the establishment of permanent endosymbiotic relationships. By employing a multidisciplinary approach, we have uncovered how the symbiotic bacterium regulates the oxidative metabolism of the trypanosomatid, integrating glucose catabolism and optimizing energy production. Our discoveries have broad implications for understanding the metabolic integration of organelles, such as mitochondria and glycosomes, with the bacterial endosymbiont. Beyond unravelling the complexities of metabolic adaptations during symbiosis, our work may contribute to the general understanding of the evolutionary dynamics of parasitism within the Trypanosomatidae family.

## INTRODUCTION

There are seven species of trypanosomatids that have coevolved with a single symbiotic bacterium and are members of the Strigomonadinae subfamily, which includes the *Angomonas*, *Kentomonas*, and *Strigomonas* genera (1, 2). These mutualistic relationships are characterized by gene transfers, structural adaptations, and metabolic exchanges, making them exceptional models for studying the origin of organelles and cellular evolution (2, 3). The symbiont origin is monophyletic, and the ancestral bacterium is phylogenetically related to gram-negative β-Proteobacteria of the Alcaligenaceae family (1, 4, 5).

The symbiosis of trypanosomatids is considered mutualistic since aposymbiotic protozoa that are obtained after antibiotic treatment are unable to colonize the intestines of host insects. These cells require nutritional supplementation for *in vitro* maintenance and are valuable models for investigating the role of the symbiont in the host metabolism (6–10). Isolated symbionts survive for up to 4 h and can synthesize proteins and phospholipids (11, 12).

The inability of symbionts to autonomously divide is related to the loss of essential bacterial division genes, whereas the host synthesizes components of the replication machinery, such as a dynamin-like protein (13–15). A hallmark of this symbiosis is the synchronization of symbiont replication with the host cell cycle. This coordination ensures that each newly generated protozoan contains one copy of essential structures, including the symbiont, nucleus, kinetoplast (a structure that contains the mitochondrial genome), and flagellum.

Mutualistic relationships assume structural and functional adaptations in associated beings. Compared with other trypanosomatids, symbiont-harboring species present a reduced paraflagellar structure, a greater distance between subpellicular microtubules, and a looser mitochondrial DNA arrangement. Conversely, symbionts have degenerated cell walls and reduced, but highly functional genomes (1, 14, 16–18). Compared with other trypanosomatids, metabolic exchange between partners increases host growth efficiency, resulting in a shorter generation time and lower nutritional requirements (19, 20). The symbiont produces enzymes and precursors for amino acid, vitamin, and heme biosynthesis, whereas the host provides essential phospholipids, such as phosphatidylcholine (5, 12, 21–24).

Symbiosis influences host mitochondrial respiration and ATP production. Ultrastructural studies have revealed close associations between the symbiont and energy-related organelles, such as the mitochondria and glycosomes. It has been suggested that symbionts optimize energy metabolism and utilize glycolytic intermediates to synthesize their carbon skeleton. In aposymbiotic cells, inhibition of oxidative phosphorylation leads to increased glycerol release and slightly reduced ATP levels, highlighting the role of the symbiont in maintaining energy efficiency (9, 10, 25, 26).

The relationship between glycosomes and the single ramified mitochondrion of trypanosomatids, such as those from the *Trypanosoma* and *Leishmania* genera, increases glucose metabolism. Proline catabolism in trypanosomatids has unique features, and it occurs completely within mitochondria and generates intermediates that are crucial for central metabolism. Insects serve as reservoirs for species such as *Leishmania*, *Crithidia*, and *Trypanosoma*, and proline, which is abundant in hemolymph, is their primary energy source (27, 28).

Trypanosomatids exhibit efficient ATP synthesis and enhanced oxidative phosphorylation, although the Krebs cycle is not used for the complete oxidation of glucose or proline but rather for the degradation of proline to succinate. In this way, procyclic *T. brucei* relies on the Krebs cycle for specific catabolic and anabolic functions, excreting succinate derived from malate or α-ketoglutarate derived from proline under glucose-rich conditions. This excretion reflects evolutionary adaptations to parasitism and host metabolic dependency (29–33).

This study investigated glucose and proline catabolism in *Angomonas deanei* and the contribution of the symbiont to these pathways. The growth, viability, oxygen consumption, and ATP production of protozoa that were cultivated with these carbon sources were evaluated. Ultrastructural analyses, nuclear magnetic resonance (NMR), and proteomics were performed to identify excreted metabolites and elucidate the metabolic landscape of *A. deanei*. Our findings revealed new aspects of metabolic coevolution between the bacterium and the protozoan host, providing insight into organelle evolution in eukaryotes as well as parasitism evolution in trypanosomatids.

## MATERIALS AND METHODS

### Cell culture

Wild-type (AdWt) (ATCC PRA-265) and aposymbiotic (AdApo) (ATCC 30969) strains of *A. deanei* were grown at 28°C in Warren’s complex medium (3.7% Sigma-Aldrich BHI broth, brain and heart infusion) (34), containing 0.1% folic acid, 25 µg/mL hemin, plus 10% supplementation with fetal bovine serum (FBS) (Vitrocell, Embriolife, Brazil) for 24 h, which corresponds to exponential cell growth. Cells were also cultivated in a chemically defined medium, as SDM80 or SDM79-CGGGPPTA Powder (GE Healthcare, Chicago, IL, USA), a variation of SDM79 medium (35) with 8 mM glucose or L-proline.

### Cell growth and viability

Cells were previously grown in Warren medium containing 10% fetal bovine serum (FBS) for 12–24 h. Subsequently, 1 × 10^6^ cells mL^−1^ were added in a fresh medium with 10% FBS (control condition), SDM80 without carbon source or FBS, SDM80 with glucose (8 mM), and SDM80 with proline (8 mM). To evaluate proliferation, cells were collected every 12 h up to 72 h and counted using a BD Accuri C6 cytometer (Accuri Cytometers Inc/Becton Dickinson, Franklin Lakes, NJ, USA). Three independent experiments in duplicate were performed. The viability assays were performed in a 96-well flat-bottom plate (NEST) with propidium iodide (20 µg/mL). Subsequently, the plate was incubated at 28°C in the dark for 15 min. A total of 10,000 protozoa were quantified by flow cytometry using a BD Accuri C6 cytometer (Accuri Cytometers Inc/Becton Dickinson, Franklin Lakes, NJ, USA), analyzing the 546 nm wavelength. Three independent experiments in triplicate were performed.

### Glucose consumption

Protozoa were grown in Warren complex medium supplemented with 10% FBS at 28°C for 24 h, and then 1 × 10^7^ cells mL^−1^ was added to fresh Warren medium supplemented with 20 mM glucose. Aliquots of 100 µL were collected after 10 min, and the cells were centrifuged at 3,000 × *g* for 1 min. The supernatant had its glucose content quantified using the Glucose GOD-PAP kit (Labtest, SA). The reaction was read with the optical density at 505 nm in a BioTek Synergy H1 Multimode Reader (Agilent, Santa Clara, CA, United States).

### ATP quantification

For these assays, 1 × 10^7^ cells mL^−1^ were washed and incubated for 30 min in 1 mL of PBS pH 7.2, containing or not containing KCN (0.25 mM). After incubation, the cells were centrifuged at 3,000 × *g* for 5 min at room temperature and resuspended in 1 mL of boiling water. Immediately after, the cells were homogenized in a vortex and incubated on ice and then centrifuged for 3 min at 12,000 × *g* at 4°C (36). The supernatant had its ATP content quantified using the ATP Life Technology Kit (USA) by analyzing luminescence in a Perkin Elmer Victor X2 luminometer (USA).

### High-resolution respirometry

In fasting assays, the cells were washed and incubated in PBS pH 7.2 for 3 h. Subsequently, 1 × 10^7^ cells mL^−1^ was taken to the oxygraphy (Oxygraph-2K, OROBOROS Instruments, Innsbruck, Austria) for the analysis of O_2_ consumption under two conditions: 8 mM glucose and 8 mM proline. The substrates were added in the course of the assay, and the analyses were performed every 45 min in three steps: first, the cells were maintained in routine respiration, and then carbonyl cyanide p-trifluoromethoxyphenylhydrazone (FCCP) (Sigma-Aldrich) was added every 1 µM to reach maximum respiration after mitochondrial uncoupling (average final concentration of 8µM). Finally, 500µM potassium cyanide (KCN) (VETEC) was added. The assays were analyzed using DatLab 5.1 Oroboros instruments software.

### Nuclear magnetic resonance (NMR)

The nuclear magnetic resonance analyses were conducted at the National Center of Nuclear Magnetic Resonance, Jiri Jonas at UFRJ, in an 800 MHz Bruker spectrometer at 25°C using a 5 mm TXI probe. We performed 1D-^1^H, 1 H-1H TOCSY, and ^1^H/^13^C HSQC spectra. The spectra acquisitions were standardized. Topspin (Bruker Biospin program, Rheinstetten, Germany) was used for data acquisition, and the MestReNova version 6.0 was used for processing the NMR spectra. One-dimensional ^1^H spectra were used for quantification, and peaks were integrated and compared with the internal reference (maleic acid). Assignments were performed using NMR data from the literature and confirmed using ^1^H-^1^H TOCSY and ^1^H-^13^C HSQC spectra.

### NMR sample preparation

Metabolic products labeled with proton (^1^H) and carbon (^13^C) that were released in the culture medium were identified by NMR analyses as previously described (37). Sample preparation for NMR quantification of the end-products was obtained using 4 × 10^8^ cells of both strains of *A. deanei*, grown in Warren medium with 10% FBS (38). When in the exponential growth phase, the cells were washed in PBS pH 7.2 and incubated for 6 h at 28°C in 5 mL of PBS pH 7.2 containing different nutrient sources at 4 mM concentration: glucose, glycerol, lactate, threonine, leucine, tryptophan, phenylalanine, histidine, arginine, isoleucine, valine, and proline. After 6 h of incubation, the samples were centrifuged at 1.500 × *g* for 10 min, and 1 mL of the supernatant was collected and frozen at −20°C for future evaluation. The samples were thawed just before NMR analyses. The NMR samples were prepared with 600 µL of the extract; 10% D_2_O and maleic acid were used as a reference. The cell viability was monitored by observing protozoa motility using optical microscopy.

### Sample preparation for mass spectrometry

Protein samples obtained from the total cell extracts of wild-type and aposymbiotic strains of *A. deanei* were separated by migration on a 10% acrylamide SDS-PAGE gel. Proteins were detected by colloidal blue staining. The gels were decolorized in a solution containing (25 mM ammonium bicarbonate [NH_4_HCO_3_], acetonitrile, 50% ACN) and dehydrated in ACN for 10 min. Afterward, the gels were dried at room temperature. Proteins were reduced in a solution containing 10 mM dithiothreitol and 100 mM NH_4_HCO_3_ for 30 min at 56°C. Then, parts of the gel were alkylated and dehydrated in 100 mM iodoacetamide and 100 mM NH_4_HCO_3_ for 30 min at room temperature and incubated in ACN for 10 min. After removing the ACN, gels were rehydrated twice with 100 mM NH_4_HCO_3_ for 10 min at room temperature. Proteins were digested by incubating gels in a solution containing 10 ng/µL trypsin (T6567, Sigma-Aldrich), 40 mM NH_4_HCO_3_, and 10% ACN. After rehydration for 10 min, the gels were incubated in water overnight at 37°C. The peptides were extracted from the gel as follows: incubation in a solution containing 40 mM NH_4_HCO_3_ and 10% ACN for 15 min at room temperature, and then two incubations in a solution containing 47.5% ACN and 5% formic acid for 15 min at room temperature. The extraction process was repeated three times, and the collected extracts were mixed with the supernatant from the initial digestion. Peptides were dried in a SpeedVac and resuspended with 25 µL of 0.1% formic acid before being subjected to nanoLC-MS/MS mass spectrometry analysis.

### NANOLC-MS analysis (LABEL-FREE)

Nano-LC-MS/MS analyses were performed using the Ultimate 3000 system (Dionex) coupled to the LTQ Orbitrap XL nanospray mass spectrometer (Thermo Fisher Scientific). For these assays, 10 µL of each peptide extract was loaded onto a column with an internal diameter (ID) of 300 µm × 5 mm length (LC Packings, Dionex) with a flow rate of 20 µL/min. After 5 min of desalting, peptides were separated on a 75 µm × 15 cm length ID C18 PepMap column (LC Packings, Dionex) with a linear gradient of 2%–40% solvent B (0.1% formic acid in 80% ACN) for 108 min. The separation rate was set at 200 nL/min. The mass spectrometer operates in positive ion mode with a needle voltage of 1.8 kV and a capillary voltage of 42 V. Data were acquired in data-dependent mode, alternating between FTMS scanning in the range 300–1,700 m/z with a resolution set at 60,000 for 400 m/z and six ion trap MS/MS scans with a CID (Collision Induced Dissociation) activation mode.

### Quantitative data analysis by LABEL-FREE in PatternLab for proteomics (PLP)

Raw LC-MS/MS data were imported into PatternLab for Proteomics software v4.1.0.11 for processing and quantification (39). The “target-decoy” database was prepared based on the protein sequences from UniProt, selecting the *Homo sapiens* organism on July 25, 2018. The final database used by the PSM contained 190,404 sequences, plus 127 sequences of contaminants most common in mass spectrometry (such as keratin, trypsin). The mass spectrometer program used was the Comet 2016 rev 3 search engine (40); the search was limited to tryptic and semi-tryptic candidates, carbamidomethylation was imposed as a fixed modification, and methionine oxidation was the variable modification. Sequences with a tolerance of 40 ppm from the precursor m/z measurement were accepted, and Xcorr and Z-Score were used as primary and secondary similarity metrics. The inherent search considered the 40 ppm mass-tolerant precursor for MS/MS as the precursor. The validity of a peptide spectrum was evaluated using PatternLab’s Search Engine Processor (SEPro) module (41) to accept a false discovery rate (FDR) of 1% based on the number of decoys. Finally, identifications diverging by more than 10 ppm from the theoretical mass or protein identified by a single spectrum with an Xcorr less than 2.5 were disregarded. A Venn diagram was generated comparing the proteins detected in each condition and using wild-type and aposymbiotic strains of *A. deanei*. UniProt protein sequences were analyzed in the TriTrypDB Database. Proteomic analysis of the *A. deanei* symbiont was carried out using proteome data (15).

### Scanning electron microscopy (SEM)

Both strains of *A. deanei* were grown in Warren’s complex medium with 10% SFB or in SDM80 medium supplemented with proline, glucose, or even in the absence of substrates for 24 h. After washing in PBS pH 7.2, the cells were deposited on Poly-L-lysine (0.1%)-coated coverslips and fixed for 30 min in 2.5% glutaraldehyde type II (EMS) diluted in 0.1 M sodium cacodylate buffer. Subsequently, the cells were washed and post-fixed in the dark in a solution containing 1% osmium tetroxide diluted in 0.8% potassium ferrocyanide and 5 mM calcium chloride for 45 min. Post-fixation, the samples were washed and subsequently dehydrated in an increasing series of ethanol (50, 70, 90, and twice at 100%) for 10 min at each step. Then, ethanol was replaced by CO_2_ for sixteen cycles in a critical point apparatus (Bal-tec CPD 030). The samples were metallized with gold (FL-9496 Balzers) before observation in an EVO MA10 Zeiss operating at 15 kV or an FEI Quanta-250 operating at 20 kV. The morphometric analysis was performed by quantifying 100 cells from each condition using software AxioVision 4.8.2 SP2 (06-2012) developed by CARL ZEISS.

### Transmission electron microscopy (TEM)

Both strains of *A. deanei* were grown in the different media as mentioned above for 24 h and, after washing in PBS pH 7.2, were fixed with 2.5% glutaraldehyde type II (EMS) diluted in 0.1 M sodium cacodylate buffer for 1 h. Subsequently, the cells were washed in the same buffer and post-fixed in the dark for 45 min in 0.8% potassium ferrocyanide solution containing 5mM calcium chloride and 1% osmium tetroxide diluted in 0.1 M sodium cacodylate buffer. The cells were washed and dehydrated in increasing concentrations of acetone (50%, 70%, 90%, and twice in 100%), 10 min at each step. Then, the samples were infiltrated for 12 h in epoxy resin (Polybed) diluted in acetone 100% (1:1), in pure resin for 6 h, and polymerized at 60°C for 72 h. Ultrathin sections (70 nm) that were obtained on a LEICA UC6 EM ultramicrotome were collected on 300 mesh copper grids and contrasted in 5% aqueous uranyl acetate solution for 45 min and lead citrate solution for 5 min in the dark. Samples were observed on a FEI TECNAI SPIRIT transmission electron microscope operating at 80 kV.

### Statistical analysis

GraphPad Prism software version 9.5.1 (GraphPad software, USA) was used to plot the graphs and calculate the means and standard errors (±). One-way and two-way ANOVA with Holm-Šídák multiple comparisons and unpaired *t*-test were also used.

## RESULTS

### Restrictive nutritional conditions affect cellular proliferation mainly in aposymbiotic protozoa

**Fig 1:**
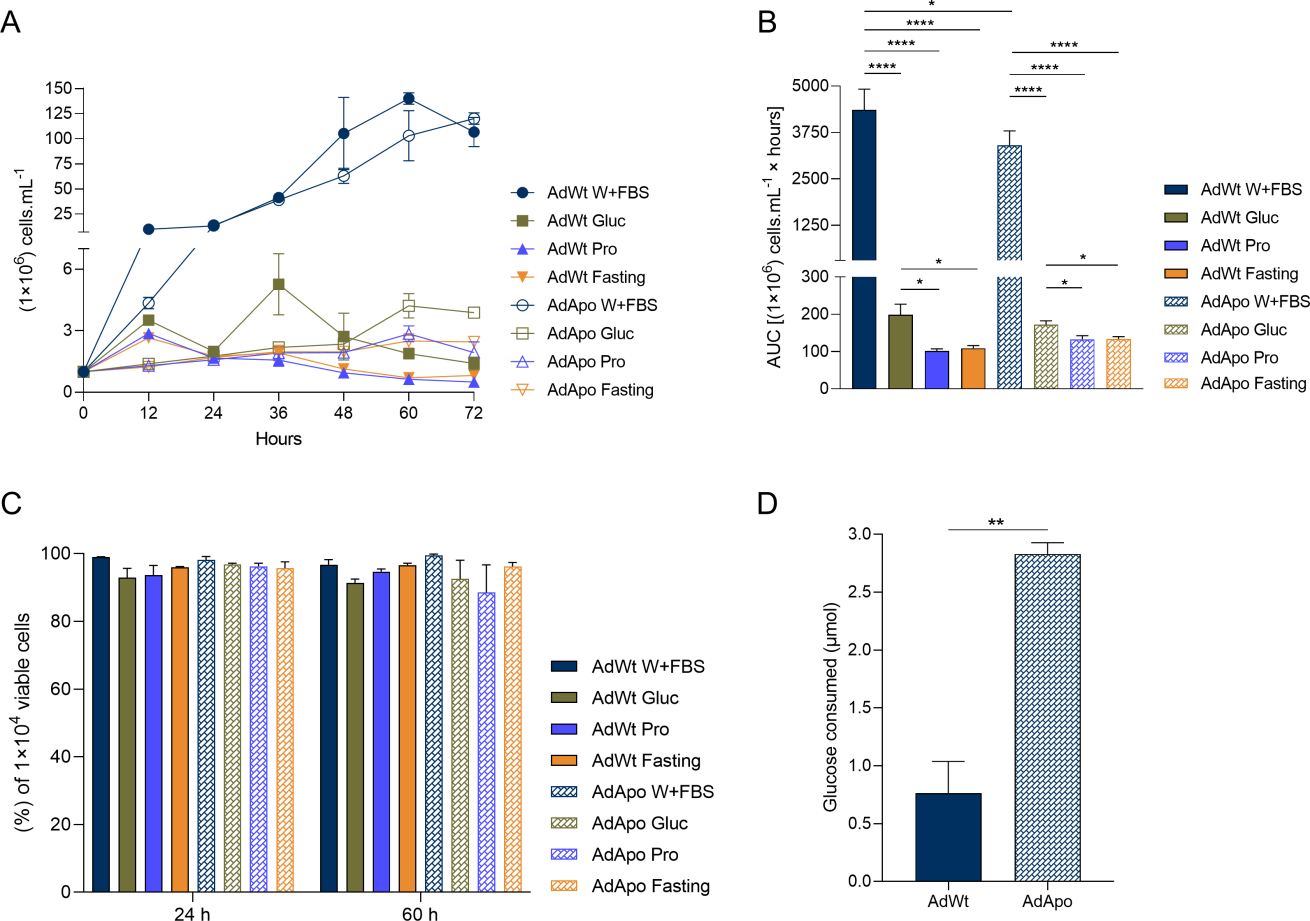
Proliferation, viability, and glucose consumption of *A. deanei*. (**A**) Proliferation curve of wild-type (AdWt) and aposymbiotic (AdApo) strains. AdWt in Warren with FBS (control), blue line and bar (●), AdWt in SDM80 medium with glucose, brown line and bar (■), AdWt in SDM80 medium with proline, light blue line and bar (▲), AdWt in SDM80 medium – fasting, orange line and bar (▼), AdApo in control medium, blue patterned line and bar (○), AdApo in SDM80 medium with glucose, brown patterned line and bar (□), AdApo in SDM80 medium with proline, light blue patterned line and bar (Δ), AdApo in SDM80 medium, fasting, orange patterned line and bar (∇). (**B**) Area under the curve of proliferation. (**C**) Cell viability after cultivation for 24 and 60 h in different nutritional conditions. (**D**) Glucose consumption in Warren medium supplemented with 20 mM glucose. Means ± SEM and statistical analyses, area under the curve for (**A**), (**B**) one-way ANOVA with Holm-Šídák’s multiple comparisons test. (**D**) Unpaired *t*-test. *P*-value **** (<0.0001), ** (<0.005), * (<0.05). (**A–D**) *N* = 3.

AdWt and AdApo strains were cultivated under various nutritional conditions for 72 h. When grown in Warren medium containing serum (control), both strains exhibited exponential growth, but the AdWt strain proliferated more robustly, 10-fold within 12 h, whereas the AdApo population increased by only 4-fold. Under fasting conditions or when grown in SDM80 medium with a single carbon source, proliferation was significantly lower than that under the control condition. AdWt cells grew exponentially for 36 h in glucose-only medium but reached a lower cell density under these conditions than under the control condition, whereas prolonged exponential growth (up to 60 h) occurred only in Warren medium supplemented with serum. AdApo cells displayed a distinct pattern in glucose-only medium, showing a growth plateau until 48 h, followed by minimal proliferation, which differed from the earlier decline observed in AdWt cells (Fig. 1A).

Area under curve analysis revealed greater values for AdWt cells than for AdApo cells under all growth conditions, as revealed by the percentage values and averages obtained when considering the different culture media. The AdWt strain that was cultivated with glucose presented areas that were 49.09% (97.6 ± 24.31) and 45.22% (89.9 ± 24.31) larger than those observed when the strain was cultivated under the proline-only and fasting conditions, respectively. AdApo cells that were cultured with glucose presented greater proliferation than those that were cultured with proline (22.95%, 39.5 ± 13.2) or under fasting conditions (22.66%, 39 ± 13.2) (Fig. 1B). The cell viability remained high (close to 100%) across all the growth conditions after 24 and 60 h (Fig. 1C). The amount of glucose consumed by the AdWt strain was 73% (2.07 ± 0.3) lower than that of the AdApo strain (Fig. 1D).

These findings highlight the dependence of AdApo cells on nutrient-rich conditions and their reduced adaptability compared with that of AdWt cells, emphasizing the metabolic contributions of the symbiotic bacterium.

### Morphological changes in *A. deanei* cultivated under nutritional restriction

**Fig 2:**
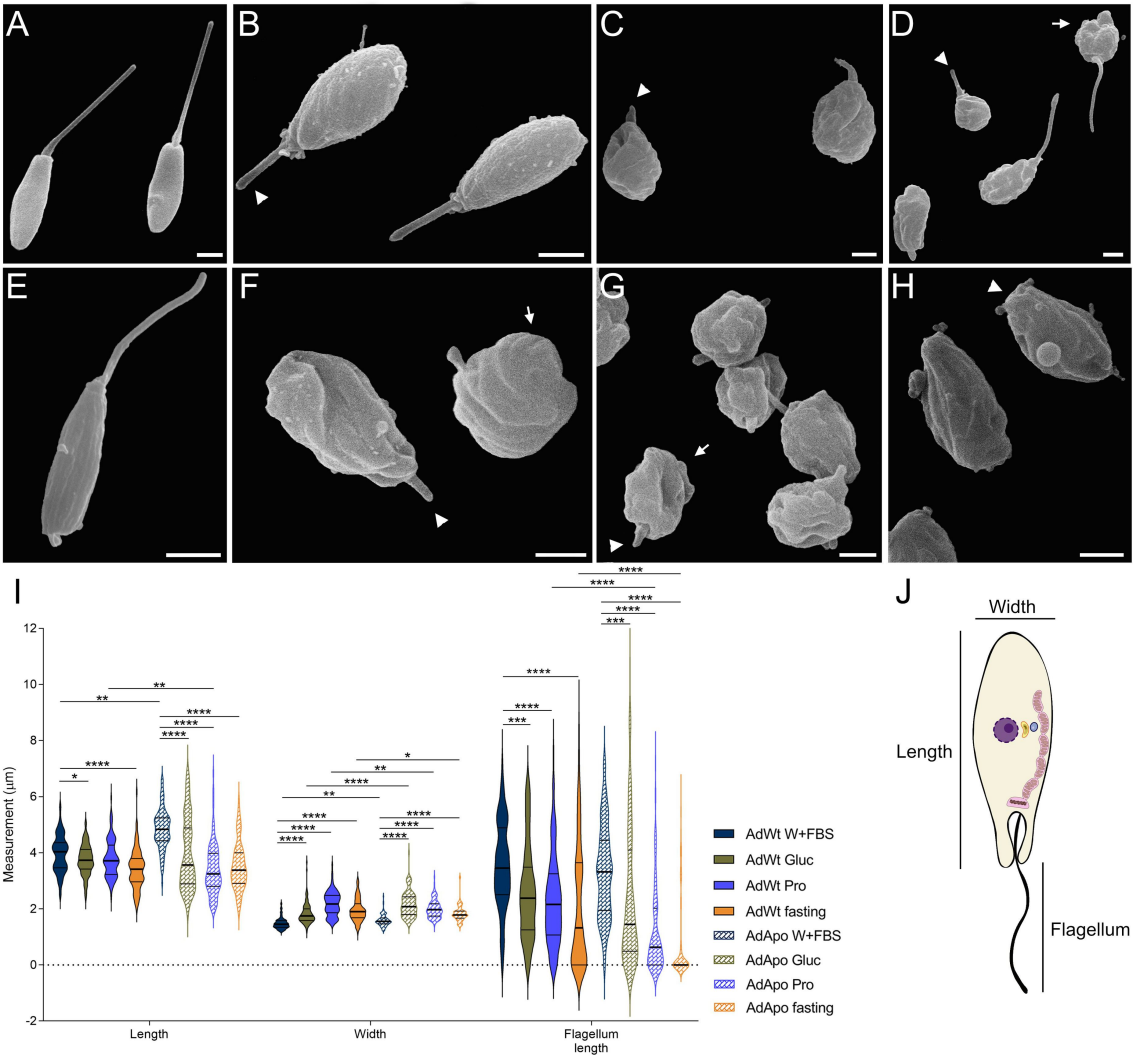
Morphological analysis of *A. deanei* cultivated in different culture media. (**A–D**) Wild-type strain (AdWt): (**A**) control medium, (**B**) SDM80 with glucose, (**C**) SDM80 with proline, (**D**) fasting. (**E–H**) Aposymbiotic strain (AdApo): (**E**) control medium, (**F**) SDM80 with glucose, (**G**) SDM80 with proline, (**H**) fasting. White arrows indicate rounded cells or protozoa with a reduced length of the cell body presenting a wrinkled surface. White arrowheads indicate flagellar shortening. (**I**) Morphometric data based on SEM analyses. (**J**) Parameters for morphometric analysis. Medians and quartiles were calculated, and statistical analyses were performed using a two-way ANOVA and a Holm-Šídák multiple comparisons test for comparisons between conditions and a one-way ANOVA for comparisons between strains. *P*-value **** (<0.0001), *** (<0.001), ** (<0.005), * (<0.05). *N* = 100 cells. Scale bars: 1 µm.

Scanning electron microscopy was used to analyze the occurrence of atypical phenotypes in *A. deanei* cells that were cultivated with a single carbon source or under fasting conditions. Both the AdWt and AdApo strains maintained their characteristic choanomastigote shape and single flagellum when grown in control medium (Fig. 2A through E). However, when grown in SDM80 medium supplemented with glucose (8 mM), both strains presented reduced, rounded cell bodies and shortened flagella (Fig. 2B through F). These morphological changes were more pronounced when cells were grown in the presence of proline (Fig. 2C through G) or under fasting conditions (Fig. 2D through H), and wrinkling of the cell surface was observed, particularly in AdWt cells.

Morphometric analysis revealed significant changes in both strains compared with the control. AdWt presented a 13.63% (0.54 ± 0.085) reduction in cell body length under fasting conditions, whereas the width increased by 23.92% (0.36 ± 0.04), 47.23% (0.7 ± 0.05), and 31.33% (0.46 ± 0.05) under glucose, proline, and fasting conditions, respectively. Flagellum length decreased by 26.34% (0.93 ± 0.24), 33.82% (1.2 ± 0.23), and 39.21% (1.4 ± 0.27), respectively, under the same conditions (Fig. 2I).

AdApo cells presented more pronounced reductions in cell body length, particularly when cultured with proline (29.5%, 1.43 ± 0.12) or in the absence of carbon sources (27.3%, 1.32 ± 0.11). The width of the AdApo cells changed less than that of the AdWt did, except under glucose conditions, whereas a marked reduction in flagellum length was noted compared with that of both the control and wild-type cells (Fig. 2I).

A comparative analysis of the two strains revealed a significant decrease in cell body length of AdApo cells, particularly when cultivated in proline-containing medium (10%, 0.38 ± 0.11) or under conditions of fasting. Reduction of width was also noted in AdApo cells grown with proline (8.4%, 0.18 ± 0.05) and in fasting conditions (6.1%, 0.12 ± 0.05). A decrease in the flagellum size was also observed: 47.8% (1.12 ± 0.24) when proline was the carbon source and 57.8% (1.25 ± 0.27) in fasting.

These data indicate that both strains undergo morphological changes when grown in SDM80 medium, more accentuated in the presence of proline or under fasting conditions, with the AdApo strain being more affected.

### Ultrastructural analysis of *A. deanei* cultivated in a single carbon source or under fasting: impact on mitochondria and glycosomes

**Fig 3:**
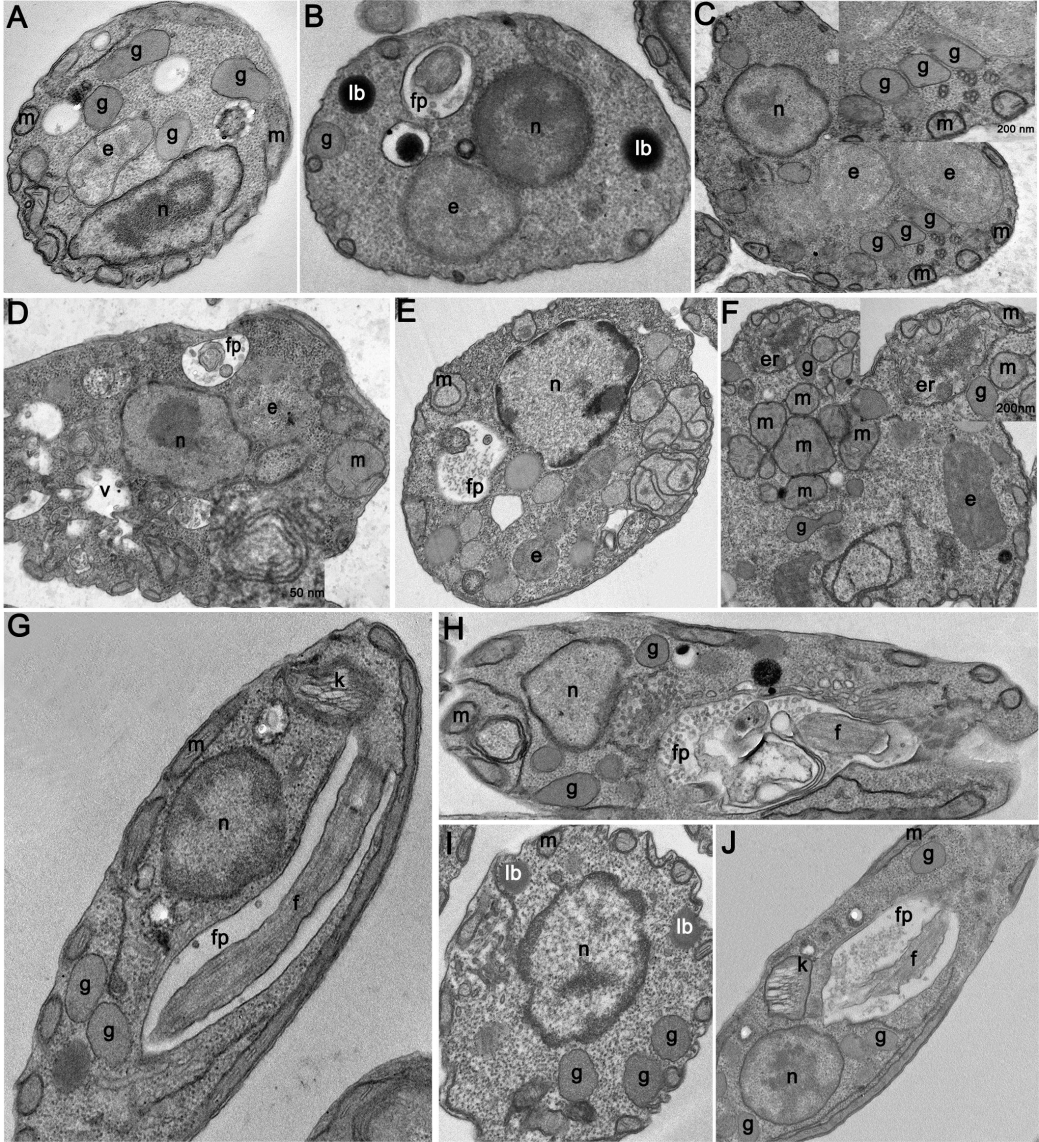
Ultrastructural analysis of *A. deanei* by TEM. (**A–F**) Wild-type strain. (**G–J**) Aposymbiotic strain. (**A**) Warren medium containing FBS. (**B**) SDM80 medium without a carbon source. Black arrows indicate mitochondrial reduction. (**C, D**) SDM80 medium with glucose. (**C**) Note the increased number of glycosomes and the fragmentation of mitochondria in the inset (black arrows). (**D**) Observe the vacuolated cytoplasm and the presence of concentric myelin figures (arrow and inset); (**E, F**) SDM80 medium with proline. (**E**) Regarding the enlargement of the mitochondrial cristae (black arrowheads) and glycosome leakage (black arrows). (**F**) Note the enlargement of mitochondrial profiles and the inset showing the endoplasmic reticulum (arrows) next to the fragmented mitochondrion (arrowhead). (**G**) Cell cultivated in Warren medium. (**H**) SDM80 medium with glucose. Note the concentric membranes close to the mitochondria (black arrow) and microvesicles in the cytosol and in the flagellar pocket (white arrow), where membrane profiles were also observed; (**I**) SDM80 medium with proline. Note lipid bodies and a possible increase in the number of glycosomes. (**J**) SDM80 without carbon sources did not present ultrastructural alterations. fp, flagellar pocket; lb, lipid body; er, endoplasmic reticulum; g, glycosome; m, mitochondria; n, nucleus; e, endosymbiont; f, flagella; k, kinetoplast. Scale bars: 500 nm.

Transmission electron microscopy was used to examine ultrastructural changes in *A. deanei* strains, with a focus on the associations of the symbiont with organelles such as the mitochondria and glycosomes. After 24 h in control medium, the AdWt cells displayed a characteristic ultrastructure, with mitochondrial branches adjacent to the plasma membrane and symbiotic bacteria near the nucleus and glycosomes (Fig. 3A). Under fasting conditions, the cells presented reduced numbers of glycosomes, smaller mitochondrial branches, and lipid bodies (Fig. 3B). When grown in SDM80 medium supplemented with glucose, the cells presented increased numbers of glycosomes, mitochondrial fragmentation, myelin figures, and cytoplasmic vacuolation (Fig. 3C through D). When the cells were cultivated in proline-supplemented medium, the mitochondrion became more centralized and presented enlarged cristae, and the proximity to the endoplasmic reticulum suggested mitophagy (Fig. 3E and F).

AdWt cells that were grown in control medium presented a typical phenotype, with glycosomes adjacent to mitochondrial branches (Fig. 3G). Under glucose conditions, the cells presented concentric membrane profiles and microvesicles in the cytoplasm and flagellar pocket, where membrane remnants, possibly related to metabolite excretion, were observed (Fig. 3H). Under proline or fasting conditions, the phenotypes of the AdApo cells resembled those of the control group (Fig. 3 and J).

These data indicate that compared with the AdApo strain, AdWt cells exhibited more pronounced ultrastructural alterations when cultured in media with a single carbon source or under starvation conditions.

### The symbiont optimizes mitochondrial physiology and energy metabolism of *A. deanei*

**Fig 4:**
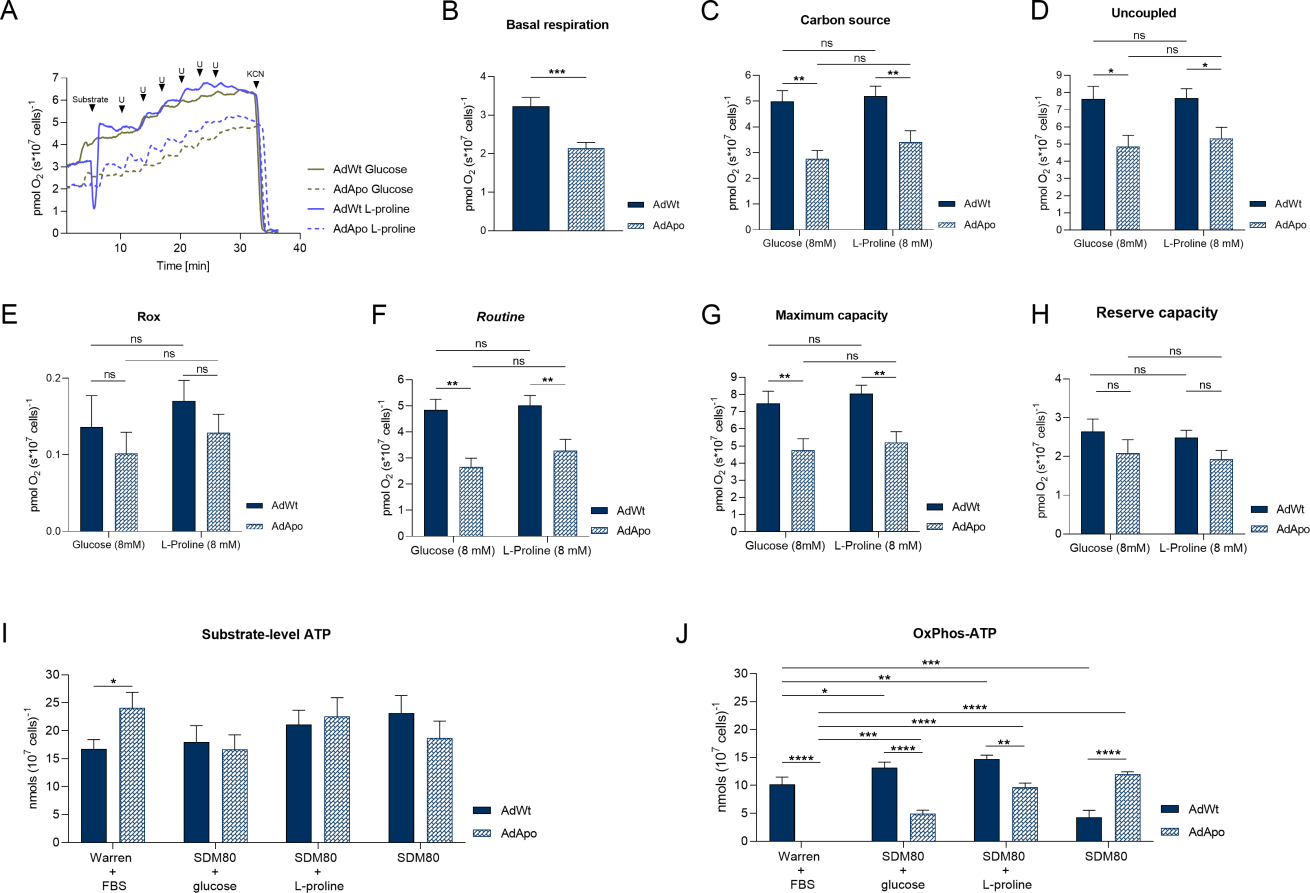
High-resolution respirometry and intracellular ATP quantification of *A. deanei*. (**A**) Representative graph of O_2_ flux per cell of wild-type (AdWt) and aposymbiotic (AdApo) strains. U indicates the addition of FCCP. (**B**) Basal respiration. (**C**) Respiration in the presence of glucose and proline. (**D**) Uncoupled respiration using FCCP. (**E**) Residual respiration (Rox). (**F**) Routine respiration. (**G**) Maximum capacity. (**H**) Reserve capacity. (**I**) ATP is produced at the substrate level after using KCN. (**J**) ATP produced by mitochondrial OxPhos. Means ± SEM and statistical analyses performed by Unpaired *t*-test (**B–I**), One-Way ANOVA (**C–H**), and two-way ANOVA with Holm-Šídák’s multiple comparisons test. *P*-value **** (<0.0001), *** (<0.001), ** (0.005), * (<0.05) (**J**).

Oxygen consumption was analyzed in AdWt and AdApo grown under conditions in which glucose or proline was used as the substrate to assess mitochondrial oxidative metabolism. Both strains presented similar respiratory profiles when cultured with these carbon sources, but the AdWt cells presented 33.68% (1.1 ± 0.27) greater basal O_2_ consumption than the AdApo cells (Fig. 4A and B). Upon the addition of glucose or proline, O_2_ consumption in AdWt was 44.48% (2.22 ± 0.56) and 34.21% (1.78 ± 0.56) greater, respectively (Fig. 4C).

The addition of 8 µM FCCP induced maximum respiration in AdWt cells grown in control conditions, whereas in proline medium, maximum respiration was achieved with 6–7 µM FCCP. Maximum respiration in AdWt cells cultured with glucose or proline was 36.32% (2.77 ± 0.91) and 30.40% (2.33 ± 0.91) greater than those in AdApo cells, respectively (Fig. 4D). The addition of KCN reduced respiration in both strains grown under all conditions, confirming that O_2_ consumption is primarily linked to oxidative phosphorylation via cytochrome c oxidase (Fig. 4E).

Compared with that in AdApo cells, routine respiration was 45.02% (2.18 ± 0.55) greater in AdWt cultured with glucose and 34.56% (1.74 ± 0.55) greater in AdWt cells cultured with proline (Fig. 4F). The maximum respiratory capacity was 63.5% (2.74 ± 0.89) and 35.15% (2.83 ± 0.89) greater in AdWt cells cultured with glucose and proline, respectively (Fig. 4G). No differences in reserve capacity were detected between AdWt and AdApo cells grown under either glucose or proline conditions (Fig. 4H).

The intracellular ATP levels were measured after 24 h of cultivation, with KCN used to inhibit mitochondrial ATP synthesis. In the control medium, the AdApo cells produced 30.4% (7.32 ± 3.03) more ATP at the substrate level than the AdWt (Fig. 4I). The OxPhos-ATP levels were 100% (12.08 ± 1.41), 62.33% (8.26 ± 1.46), and 34.38% (5.05 ± 1.46) greater in the AdWt cells grown under the control, glucose, and proline conditions, respectively. In SDM80 medium without carbon sources, OxPhos-ATP levels were 64.41% (7.76 ± 1.46) greater in AdApo (Fig. 4C).

These results suggest that the presence of the symbiont optimizes the oxidative activity. In contrast, AdApo cells rely more on substrate-level ATP synthesis and mainly on oxidative phosphorylation when nutrients are scarce.

### Metabolite excretion of the host cell is influenced by the symbiont

**Fig 5:**
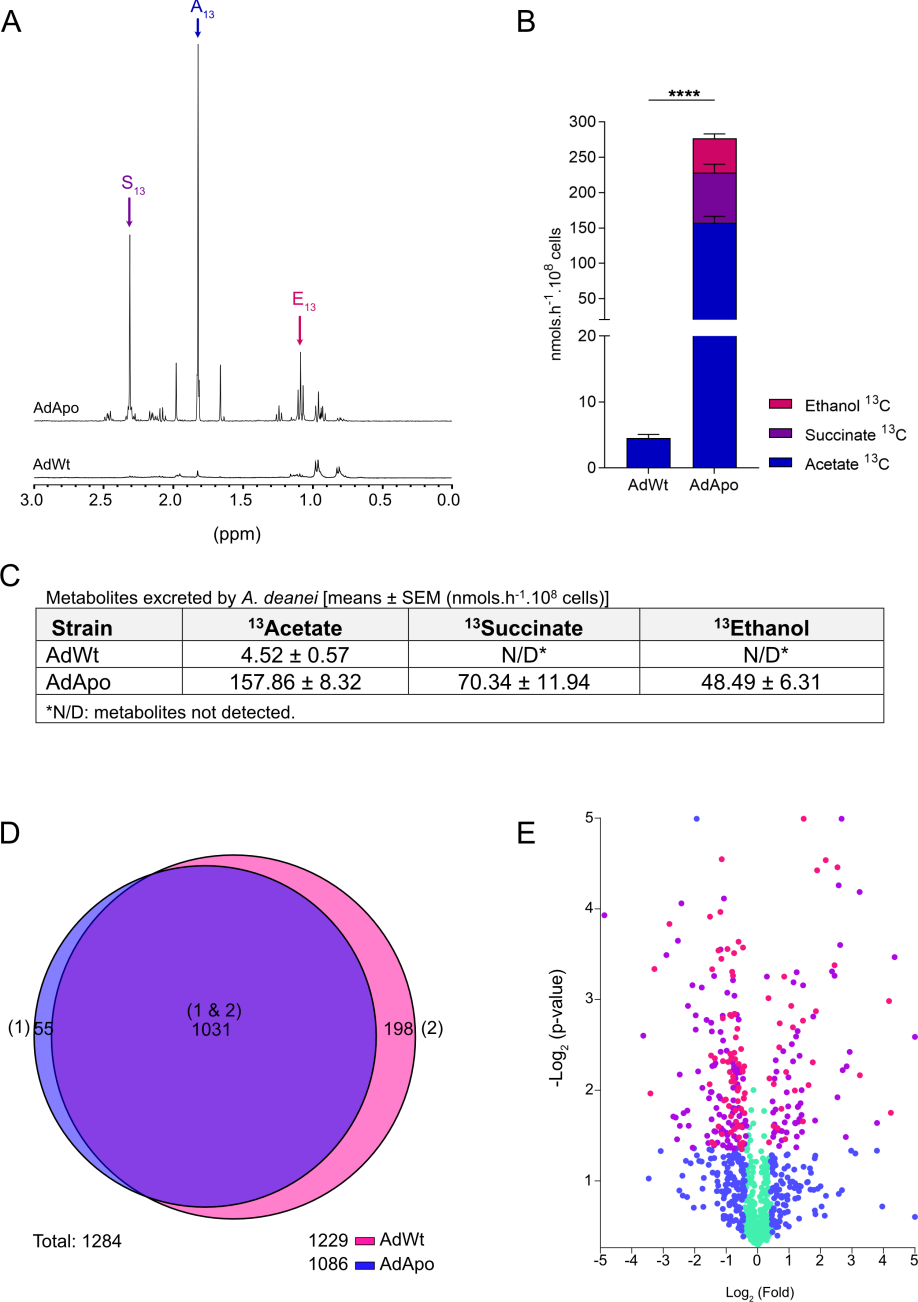
Proton (^1^H) 1D-NMR spectra and incorporation ratios of [^13^C-U]-glucose in metabolic end-products excreted and proteomic analysis of *A. deanei*. (**A**) Representative spectra of AdApo and AdWt, respectively. (**B, C**) Quantification of metabolites. E, ethanol; S, succinate; and A, acetate. (**D**) Venn diagram of the proteomics of the total extract of *A. deanei*, the AdWt (1) and AdApo (2) strains using the PLP program. This diagram shows the number of proteins found in each proteome, from the wild strain (1,229, pink) and the aposymbiotic strain (1,086, blue), the proteins common to both groups (1,031, purple) and those exclusive to the wild strain (198) or the aposymbiotic strain (55). (**E**) Comparative proteomic analysis between AdWt and AdApo strains. Pink dots: proteins with *P* Value less than 0.05; purple dots: low-abundant proteins; blue dots: proteins more abundant in one strain than the other, with low statistical power; cyan dots: proteins equally abundant in both *A. deanei* strains. (**A–C**) Means ± SEM and statistical analyses performed by Unpaired *t*-test. *P*-value **** (<0.0001). *N* = 5 to AdWt and *N* = 6 to AdApo.

To assess the carbon sources that support *A. deanei* metabolism and the influence of the symbiont, excreted products were analyzed using nuclear magnetic resonance (NMR). Cell extracts prepared with glucose, glycerol, lactate, and amino acids were quantified using maleic acid as an internal reference. Only glucose resulted in the excretion of products, which were identified as ethanol, succinate, and acetate (Fig. 5A).

To further investigate this phenomenon, U-^13^C-glucose was used to trace metabolite incorporation. The ^13^C label caused a split in the ^1^H NMR spectra, allowing comparison with ^12^C metabolites. Metabolite end product levels in the AdWt strain were 146.6 times lower than those in the AdApo. AdWt cells secreted only acetate, whereas the AdApo strain excreted acetate, ethanol, and succinate (Fig. 5 and C).

These findings highlight the distinct metabolic activities influenced by the symbiont.

### Proteomic analysis: how the symbiont integrates the metabolic pathways of *A. deanei*

Comparative proteomic analyses were conducted to determine the impact of the symbiont on the biosynthetic pathways and energy metabolism of *A. deanei*. A total of 1,284 proteins were identified, with 1,229 in the AdWt strain, 1,086 in the AdApo, and 1,031 shared between the two strains. Notably, 198 proteins were unique to the AdWt strain, and 55 were unique to the AdApo (Fig. 5D).

**Fig 6:**
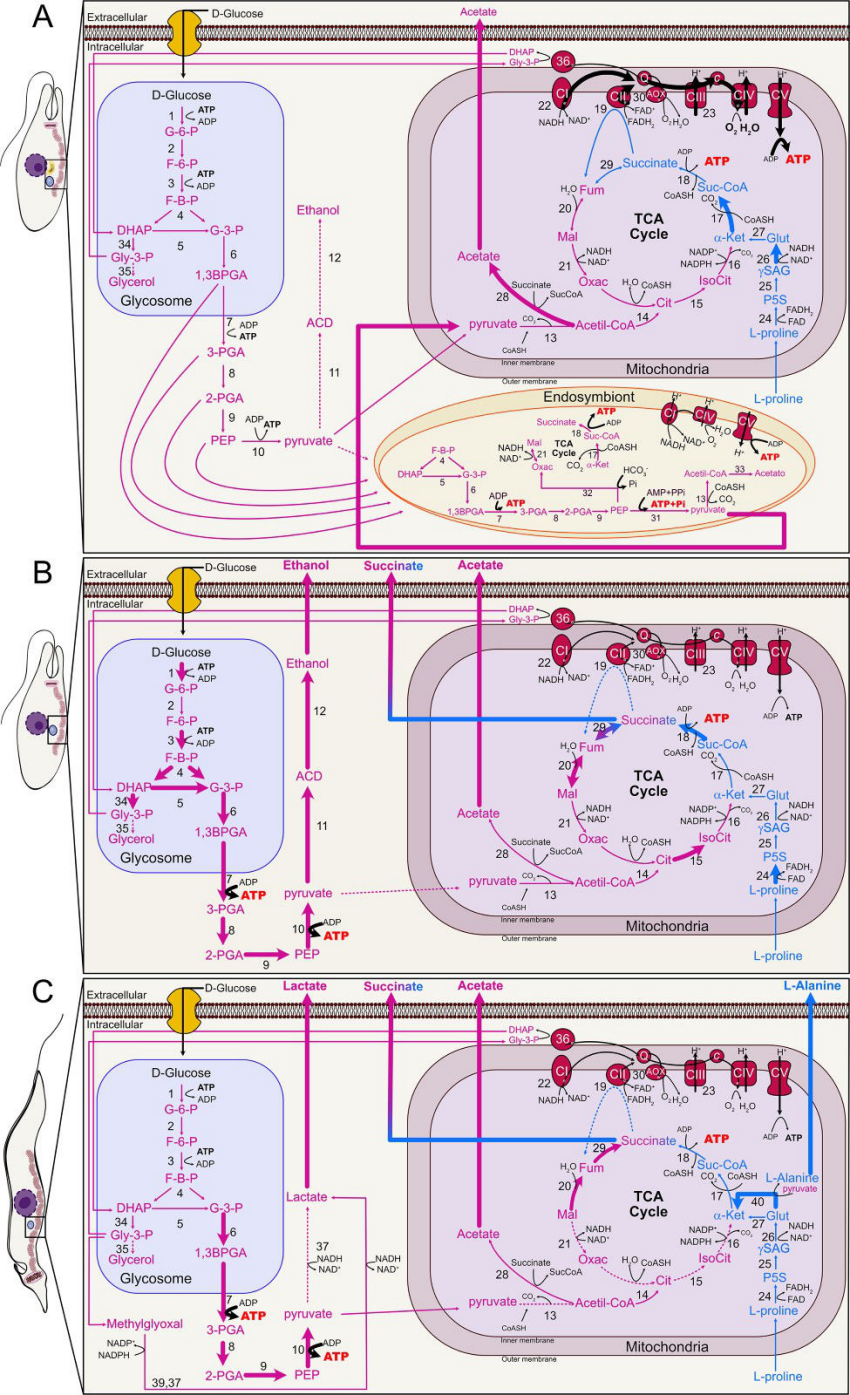
Schematic representation illustrates glucose and L-proline metabolism in wild-type (**A**), aposymbiotic (**B**) *A. deanei* strains, and the procyclic form of *T. brucei* (**C**) adapted (30). This diagram provides a detailed overview of metabolic pathways and their enzymatic steps in these organisms. Pink arrows indicate glucose metabolism, whereas blue arrows represent L-proline metabolism. Excreted end products are displayed in the extracellular region: acetate for the wild-type strain (AdWt) and alcohol, succinate, and acetate for the aposymbiotic strain (AdApo). Reversible reactions are depicted only in their presumed or demonstrated direction, and dashed arrows indicate steps occurring at background levels. The schematic highlights key organelles, including the glycosome and mitochondria, along with the tricarboxylic acid (TCA) cycle. Abbreviations for metabolites include: G-6-P, glucose-6-phosphate; F-6-P, fructose-6-phosphate; F-B-P, fructose-1,6-bisphosphate; DHAP, dihydroxyacetone phosphate; G-3-P, glyceraldehyde-3-phosphate; 1,3BPGA, 1,3-bisphosphoglycerate; 3-PGA, 3-phosphoglycerate; 2-PGA, 2-phosphoglycerate; PEP, phosphoenolpyruvate; ACD, acetaldehyde; Cit, citrate; IsoCit, isocitrate; αKet, α-ketoglutarate; SucCoA, succinyl-CoA; Fum, fumarate; Mal, malate; Oxac, oxaloacetate; P5C, pyrroline-5-carboxylate; γSAG, glutamate-γ-semialdehyde; Q, ubiquinone pool; C, cytochrome c; and CoASH, coenzyme A. The enzymes involved are: 1, Hexokinase; 2, glucose-6-phosphate isomerase; 3, phosphofructokinase; 4, aldolase; 5, triosephosphate isomerase; 6, glyceraldehyde-3-phosphate dehydrogenase; 7, cytosolic phosphoglycerate kinase; 8, phosphoglycerate mutase; 9, enolase; 10, pyruvate kinase; 11, pyruvate decarboxylase; 12, alcohol dehydrogenase; 13, pyruvate dehydrogenase complex; 14, citrate synthase; 15, aconitase; 16, NADP-dependent isocitrate dehydrogenase; 17, α-ketoglutarate dehydrogenase complex; 18, succinyl-CoA synthetase; 19, succinate dehydrogenase (complex II); 20, mitochondrial fumarate hydratase; 21, mitochondrial malate dehydrogenase; 22, rotenone-insensitive NADH dehydrogenase; 23, Complex III; 24, proline dehydrogenase; 25, spontaneous reaction; 26, Δ−1-pyrroline-5-carboxylate dehydrogenase; 27, glutamate dehydrogenase; 28, acetate:succinate CoA-transferase (ASCT); 29, fumarate reductase; 30, alternative oxidase (AOX); 31, pyruvate dikinase; 32, phosphoenolpyruvate carboxylase; 33, acetyl-CoA synthetase; 34, glycerol-3-phosphate dehydrogenase; 35, glycerol kinase; 36, mitochondrial glycerol-3-phosphate dehydrogenase; 37, lactate dehydrogenase; 38, non-enzymatic reaction; 39, NADPH-dependent methylglyoxal reductase; and 40, L-alanine aminotransferase. Respiratory chain components include CIV, cytochrome c oxidase complex, and CV, F0/F1-ATP synthase.

Volcano plot analysis revealed that 10.5% of the proteins were significantly more abundant in one strain, with 19.6% more frequent, but not abundant, and 31.2% of the proteins were significantly less abundant (Fig. 6E). Gene ontology analysis (GO) of both *A. deanei* strains showed that 46% of the proteins present molecular function, whereas 27.2% are involved in biological processes and 26.8% in cellular components (Fig. S1). The biological process category showed the greatest variation when both strains were compared, particularly in primary metabolism, production of organic and nitrogenous compounds, biosynthesis, and catabolism (Fig. S2 and S3).

Proteomic analyses revealed marked upregulation of glycolytic enzymes in the AdApo strain, as indicated by a negative fold change in relation to AdWt. Notably, enzymes that contribute to pyruvate production presented increased expression levels, including glycosomal hexokinase (−1.48-fold), phosphofructokinase (−1.63-fold), aldolase (−1.67-fold), triosephosphate isomerase (−1.53-fold), glyceraldehyde-3-phosphate dehydrogenase (−1.34-fold), two isoforms of phosphoglycerate kinase (−1.76-fold and −1.84-fold), enolase (−2.23-fold), and pyruvate kinase (−2.54-fold) (Table 1). Furthermore, enzymes that are involved in glycerol metabolism and the alcoholic fermentation pathway, such as glycerol-3-phosphate dehydrogenase, indolepyruvate decarboxylase, and alcohol dehydrogenase, were significantly upregulated in the AdApo strain, indicating an increased reliance on fermentative metabolism in the absence of the symbiont.

**TABLE 1:** Differentially expressed proteins in wild-type and aposymbiotic strains of *A. deanei*

Uniprot ID	TriTrypDB ID	Protein	Fold Change^*a*^	*P* Value	Pathway
A0A7G2CQV1	ADEAN_000840700	Glycosomal Hexokinase	−1.48	0.0247	Glycolysis
A0A7G2CI54	ADEAN_000613200	ATP-dependent 6-phosphofructokinase	−1.63	0.0026	Glycolysis
S9VFL2	ADEAN_000166300	Fructose-bisphosphate aldolase	−1.67	0.0014	Glycolysis
S9VCS7	ADEAN_000741400	Triosephosphate isomerase	−1.53	0.0002	Glycolysis
S9UUJ2	ADEAN_000193800	Glyceraldehyde-3-phosphate dehydrogenase	−1.34	0.0176	Glycolysis
A0A7G2C975	ADEAN_000276800	Phosphoglycerate kinase	−1.76	0.0005	Glycolysis
A0A7G2CBA0	ADEAN_000276700	Phosphoglycerate kinase	−1.84	0.0015	Glycolysis
S9UQY9	ADEAN_000687000	Enolase	−2.23	0.0004	Glycolysis
S9U × 27	ADEAN_000213600	Pyruvate kinase	−2.54	0.0050	Glycolysis
A0A7G2CGP5	ADEAN_000654100	Glycerol-3-phosphate dehydrogenase	−2.84	0.0327	Glycerol metabolism
A0A7G2C505	ADEAN_000224800	Indolepyruvate decarboxylase	−1.68	0.0265	Fermentation
A0A7G2CIJ2	ADEAN_000674100	Alcohol dehydrogenase	−1.15	0.0163	Fermentation
A0A7G2CH48	ADEAN_000664300	Alcohol dehydrogenase (NADP+)	−1.43	0.0249	Fermentation
S9UWX1	ADEAN_000020300	Alcohol dehydrogenase	−1.64	0.0045	Fermentation
A0A7G2CC06	ADEAN_000305700	Succinyl-CoA:3-ketoacid-coenzyme A transferase	2.33	0.0025	Krebs cycle
A0A7G2CVC5	ADEAN_000980200	Aconitate hydratase	−1.43	0.0438	Krebs cycle
A0A7G2BZA1	ADEAN_000028100	2-oxoglutarate dehydrogenase E1 component	1.89	0.0104	Krebs cycle
S9UNV3	ADEAN_000489100	Succinate—CoA ligase [ADP-forming] subunit alpha, mitochondrial	−1.78	0.0015	Krebs cycle
A0A7G2C976	ADEAN_000201300	NADH-dependent fumarate reductase	−1.55	0.0130	Krebs cycle
S9UBD7	ADEAN_000746800	Fumarate hydratase, class I	−2.77	0.0103	Krebs cycle
A0A7G2CKH6	ADEAN_000741800	Malate dehydrogenase (Oxaloacetate-decarboxylating)	−1.95	0.0003	Krebs cycle
A0A7G2C6C5	ADEAN_000257200	Proline dehydrogenase	−1.92	0.0048	Proline catabolism
S9VNY2	ADEAN_001053600	Delta-^1^-pyrroline-5-carboxylate dehydrogenase	2.17	0.0020	Proline catabolism
S9WM97	ADEAN_000947600	Glutamate dehydrogenase	−1.26	0.0432	Proline catabolism
S9V9W8	ADEAN_000057000	NADH dehydrogenase	−1.43	0.0437	OxPhos
A0A7G2CEZ8	ADEAN_000587800	NADH-cytochrome b5 reductase	3.60	0.0013	OxPhos
A0A7G2CKK4	ADEAN_000712600	NADH-cytochrome b5 reductase	−1.68	0.0003	OxPhos
S9V6B7	ADEAN_000015100	Cytochrome b-domain protein	−1.80	0.0288	OxPhos

^*a*^ Positive fold-change values reflect higher abundance in the wild-type strain, while negative fold-change values reflect higher abundance in the aposymbiotic strain.

The mitochondrial enzymes involved in the Krebs cycle were more expressed in the AdApo strain. Notably, 2-oxoglutarate dehydrogenase and succinyl-CoA:3-ketoacid-coenzyme A transferase9 was upregulated in the AdWt (1.89-fold and 2.33-fold, respectively), indicating that central carbon metabolism is influenced by the presence of the symbiont (Table 1).

Proteins that participate in proline catabolism showed varied expression: proline dehydrogenase and glutamate dehydrogenase were upregulated in the AdApo strain, whereas Δ-^1^-pyrroline-5-carboxylate dehydrogenase was more expressed in the AdWt (2.17-fold) (Table 1). Analysis of the electron transport chain revealed increased expression of NADH dehydrogenase (complex I) in AdApo cells and increased expression of NADH-cytochrome b5 reductase (complex III) in AdWt (Table 1).

Analyses of proteomic data from the *A. deanei* symbiont identified enzymes that are involved in glycolysis, such as aldolase, triosephosphate isomerase, glyceraldehyde-3-phosphate dehydrogenase, phosphoglycerate kinase, and enolase. Enzymes involved in pyruvate metabolism were also identified, as pyruvate carboxylase and pyruvate dikinase. Considering the Krebs cycle, the following enzymes were found: pyruvate dehydrogenase complex, aconitase, α-ketoglutarate dehydrogenase complex, succinyl-CoA synthetase, succinate dehydrogenase, fumarate hydratase, and malate dehydrogenase. Enzymes that participate in carbon catabolite repression, such as HPr kinase/phosphorylase and phosphocarrier protein, as well as enzymes involved in oxidative phosphorylation (OxPhos), were also identified (Table 2). These data together indicate that the symbiont contributes to the host metabolic integration by enhancing mitochondrial oxidation, glucose catabolism, pyruvate production, and ATP synthesis.

**TABLE 2:** Proteins of *A. deanei* symbiont

Protein ids	Protein
M1LVY7	Fructose-1,6-bisphosphate aldolase (FBP aldolase) (EC 4.1.2.13)
M1L573	Triosephosphate isomerase (TIM) (TPI) (EC 5.3.1.1) (Triose-phosphate isomerase)
M1LTJ2	Glyceraldehyde-3-phosphate dehydrogenase (EC 1.2.1.-)
M1LP11	Phosphoglycerate kinase (EC 2.7.2.3)
M1M752	2,3-bisphosphoglycerate-dependent phosphoglycerate mutase (BPG-dependent PGAM) (PGAM) (Phosphoglyceromutase) (dPGM) (EC 5.4.2.11)
M1LQ27	Enolase (EC 4.2.1.11) (2-phospho-D-glycerate hydro-lyase) (2-phosphoglycerate dehydratase)
M1LNW3	Phosphoenolpyruvate carboxylase (PEPC) (PEPCase) (EC 4.1.1.31)
M1L4J1	Phosphoenolpyruvate synthase (PEP synthase) (EC 2.7.9.2) (Pyruvate, water dikinase)
M1LWI6	Acetyltransferase component of pyruvate dehydrogenase complex (EC 2.3.1.12)
M1L4E1	Pyruvate dehydrogenase E1 component (EC 1.2.4.1)
M1LQ20	Dihydrolipoyllysine-residue succinyltransferase component of 2-oxoglutarate dehydrogenase complex (EC 2.3.1.61) (2-oxoglutarate dehydrogenase complex component E2)
M1M5R8	Succinate—CoA ligase [ADP-forming] subunit alpha (EC 6.2.1.5) (Succinyl-CoA synthetase subunit alpha) (SCS-alpha)
M1LWZ5	Malate dehydrogenase (Oxaloacetate-decarboxylating)(NADP+)
M1LQB1	NADH-quinone oxidoreductase subunit D (EC 7.1.1.-) (NADH dehydrogenase I subunit D) (NDH-1 subunit D)
M1L567	NADH-quinone oxidoreductase subunit C (EC 7.1.1.-) (NADH dehydrogenase I subunit C) (NDH-1 subunit C)
M1LUK9	NADH dehydrogenase I subunit E (EC 1.6.5.3)
M1LQA6	NADH-quinone oxidoreductase subunit I (EC 7.1.1.-) (NADH dehydrogenase I subunit I) (NDH-1 subunit I)
M1L × 64	NADH-quinone oxidoreductase subunit B (EC 7.1.1.-) (NADH dehydrogenase I subunit B) (NDH-1 subunit B)
M1M6S9	NADH-quinone oxidoreductase subunit K (EC 7.1.1.-) (NADH dehydrogenase I subunit K) (NDH-1 subunit K)
M1L557	NADH dehydrogenase I subunit M (EC 1.6.5.3)
M1L563	NADH-quinone oxidoreductase subunit H (EC 7.1.1.-) (NADH dehydrogenase I subunit H) (NDH-1 subunit H)
M1LQA0	NADH-quinone oxidoreductase subunit N (EC 7.1.1.-) (NADH dehydrogenase I subunit N) (NDH-1 subunit N)
M1L × 56	NADH dehydrogenase I subunit L (EC 1.6.5.3)
M1M765	Cytochrome bd-I oxidase subunit I (EC 1.10.3.-)
M1LXJ4	Cytochrome bd-I oxidase subunit II (EC 1.10.3.-)
M1L3P4	ATP synthase subunit beta (EC 7.1.2.2) (ATP synthase F1 sector subunit beta) (F-ATPase subunit beta)
M1LVS0	ATP synthase subunit b (ATP synthase F(0) sector subunit b) (ATPase subunit I) (F-type ATPase subunit b) (F-ATPase subunit b)
M1LNU5	ATP synthase gamma chain (ATP synthase F1 sector gamma subunit) (F-ATPase gamma subunit)
M1M5B4	ATP synthase subunit delta (ATP synthase F(1) sector subunit delta) (F-type ATPase subunit delta) (F-ATPase subunit delta)
M1LTD0	ATP synthase subunit alpha (EC 7.1.2.2) (ATP synthase F1 sector subunit alpha) (F-ATPase subunit alpha)
M1LVS4	ATP synthase epsilon chain (ATP synthase F1 sector epsilon subunit) (F-ATPase epsilon subunit)
M1L3N9	ATP synthase subunit c (ATP synthase F(0) sector subunit c) (F-type ATPase subunit c) (F-ATPase subunit c) (Lipid-binding protein)
M1M6M9	Acetyl-CoA synthetase (EC 6.2.1.1)
M1L5K4	Glycerol-3-phosphate dehydrogenase [NAD(*P*)+] (EC 1.1.1.94) (NAD(*P*)H-dependent glycerol-3-phosphate dehydrogenase)
M1L5M1	HPr kinase/phosphorylase (HPrK/*P*) (EC 2.7.11.-) (EC 2.7.4.-) (HPr(Ser) kinase/phosphorylase)
M1LPB4	Phosphocarrier protein

## DISCUSSION

Symbiotic associations give insights to better understand how interspecific and harmonious relationships led to the emergence of organelles, such as mitochondria and chloroplasts, throughout evolution (42). Trypanosomatid species, such as *A. deanei*, maintain a mutualistic relationship with a single bacterium, making them ideal models for studying cellular evolution. This relationship is supported by cellular integration, gene transfer, and intense metabolic exchanges between the associated partners (3, 14).

This study revealed that the symbiont increases the proliferative rate of *A. deanei* under different conditions. Both strains can grow in the presence of glucose, but proline supports proliferation only in aposymbiotic cells. Under the fasting condition, the growth rate of the AdWt strain is also lower than that of the AdApo strain. This is different in trypanosomatids that do not naturally harbor symbiotic bacteria, such as those from the *Crithidia*, *Leishmania*, and *Trypanosoma* genera, where proline is the primary carbon source in insect hosts (43–45). The AdApo strain exhibits higher glucose consumption compared to wild-type cells, which is consistent with proteomic and NMR analyses. This suggests that the symbiont performs metabolic integration between organelles, such as the mitochondrion and glycosomes, and that in its absence, the overflow metabolism occurs (46).

The morphology of both *A. deanei* strains was affected after growth in medium containing only glucose or proline as a carbon source, as well as in fasting conditions. Cells with loss of the choanomastigote shape and shortening of the flagellum were frequently observed. Similar morphological alterations were observed in *T. cruzi* epimastigotes under nutritional, thermal, or oxidative stress conditions, which can trigger metacyclogenesis (47–49). Wild-type *A. deanei* grown in glucose-supplemented medium exhibited ultrastructural changes indicative of nutritional stress, such as mitochondrial swelling and cristae enlargement, suggesting increased oxidative metabolism. Transmission electron microscopy images also indicated a rise in the number of glycosomes, which is a phenotype also observed in *T. brucei* under glucose-rich conditions (9, 30, 50). Additionally, high cytosolic vacuolation was observed, as previously reported in *T. cruzi* under stress and can be associated with autophagy (51, 52).

AdWt cells that were grown with proline exhibited mitochondrial redistribution to the cell center, with endoplasmic reticulum profiles involving mitochondrial fragments, which is indicative of mitophagy. This may reflect the exclusive use of proline for energy metabolism, leading to redox imbalance, as observed in *T. cruzi*, where proline metabolism primarily involves Δ-^1^-pyrroline-5-carboxylate dehydrogenase and NADPH oxidation (53). Under fasting conditions, AdWt presented fewer mitochondrial branches. AdApo cells exhibited no significant morphological alterations, except when grown in medium supplemented with glucose. Under such conditions, vesicles and membrane remnants were observed in the flagellar pocket, possibly due to excreted products.

In respirometry assays, the oxygen consumption of AdWt cells was greater than that of AdApo, supporting the idea that the symbiont influences the trypanosomatid energetic metabolism. This result is consistent with previous data showing that the bacterium can consume oxygen when isolated from the host (9, 25). In trypanosomatids, proline catabolism occurs within mitochondria, optimizing oxidative phosphorylation (30, 53). In *A. deanei*, the presence of the symbiont increases this amino acid consumption (54). The respiratory reserve capacity was similar in both strains when grown in the presence of glucose or proline, but the maximum respiratory capacity was greater in AdWt. Together, these data suggest that the symbiont increases oxidative metabolism, whereas the AdApo is more fermentative.

ATP production via mitochondrial oxidative phosphorylation (OxPhos) was significantly increased in the AdWt strain, when compared with AdApo, regardless of the carbon source. This observation highlights the essential role of the symbiont in increasing mitochondrial metabolic efficiency and supporting ATP synthesis. In the absence of the bacterium, ATP synthesis occurs primarily via glycolysis. Similar results were reported for *S. culicis*, where symbiont-harboring cells generated almost twice as much ATP in contrast to aposymbiotic protozoa (9).

In *A. deanei*, ATP production was higher in cells grown in proline-containing medium, which is consistent with proline metabolism occurring in the mitochondrion. Under fasting conditions, the AdApo strain generated more mitochondrial ATP, suggesting a reliance on OxPhos in the absence of carbon sources. In AdWt, competition between mitochondria and bacteria for substrates may occur. These data may be related to the fact that the AdApo strain presented fewer ultrastructural changes, including in the mitochondrion, when compared with the AdWt cells grown in media with a single carbon source or under starvation conditions.

Furthermore, by restricting carbon sources to glucose or proline, it was possible to observe a recovery of ATP production by oxidative phosphorylation (OxPhos) in the AdApo. Therefore, it can be proposed that under these conditions, the difference in ATP-OxPhos between the two strains is due to the symbiont ATP production. However, further studies are needed to confirm this hypothesis.

Metabolite excretion by *A. deanei* grown in medium containing glucose isotopes showed that the AdWt strain excreted only low amounts of acetate as a fermentation product. In contrast, the AdApo excreted additional metabolites, such as ethanol and succinate, supporting the idea that in the absence of the bacterium, cells are more fermentative, whereas the AdWt strain relies on oxidative metabolism. In trypanosomatids that naturally lack a symbiotic bacterium, glucose catabolism is incomplete, resulting in the generation of fermentation products such as succinate, acetate, lactate, pyruvate, and alanine (29, 55, 56). AdApo cells excreted fewer metabolic products than these trypanosomatids.

Noteworthy, except for glucose, we did not detect significant amounts of excreted metabolites by NMR using different carbon sources, reflecting the low nutritional requirements of *A. deanei*. This was predictable because the symbiotic bacterium completes several biosynthetic pathways of the host trypanosomatid, integrating the metabolism of the mitochondrion to glycosomes (6, 9, 19, 21–23). Our data indicate that the symbiont favors glucose consumption to optimize the energy metabolism of *A. deanei*.

Proteomic analysis revealed the upregulation of glycolytic and fermentation enzymes in AdApo cells compared with AdWt. The upregulation of glycolytic enzymes that are involved in fermentative metabolism has also been observed in other microorganisms, such as fungi (46), supporting the idea that the AdApo is more fermentative. Six enzymes that are involved in the Krebs cycle are highly expressed in the AdApo strain, which is consistent with the increased excretion of succinate and acetate. These findings suggest that the upregulation of these enzymes leads to the production of metabolic intermediates that must be excreted to prevent cellular toxicity (30, 53).

The expression of enzymes that are involved in proline catabolism was greater in the AdApo. Proline metabolites support amino acid synthesis, leading to increased production of Krebs cycle intermediates, such as succinate and acetate, which are subsequently excreted. The upregulation of acetate:succinate CoA-transferase, which regulates succinyl-CoA production, in the AdWt strain is consistent with cells excreting only acetate (30). The sole protein that was upregulated in the AdWt was Δ-^1^-pyrroline-5-carboxylate dehydrogenase, which plays a crucial role in redox and energy metabolism (53). Enzyme analysis of the electron transport system revealed that complex I was more expressed in the AdApo strain, whereas cytochrome c reductase b5 (alternative oxidase) was upregulated in the AdWt, which is consistent with greater oxygen consumption in symbiont-harboring cells.

Proteome data of the *A. deanei* endosymbiont exhibited a reduced enzymatic machinery in the glycolytic pathway, the pyruvate metabolism, the Krebs cycle, and OxPhos. Notably, two enzymes involved in carbon catabolite repression were identified, HPr kinase/phosphorylase and phosphotransferase protein. These enzymes modulate the preferred carbon source for energy metabolism. In *Sinorhizobium meliloti*, a symbiont of alfalfa and other legumes, the gene deletion of HPr kinase/phosphorylase enzyme reduced the bacterial proliferation and decreased differentiation into bacterioids, thus impairing nitrogen fixation and consequently the symbiotic relationship with the host (57). In *A. deanei*, considering the structural proximity between the symbiont, mitochondrion, and glycosomes, as well as metabolic differences when comparing the two strains, it can be hypothesized that the bacterium promotes carbon catabolism repression. It can also be suggested that the symbiont benefits from the metabolic intermediates of glycolysis, regulates the flow of glucose catabolism, and provides pyruvate to the mitochondrion, thus optimizing the host oxidative metabolism.

### Conclusions

In conclusion, the symbiont significantly influences the production of intermediate substrates, ensuring metabolic integration and supporting an efficient host trypanosomatid metabolism. The presence of the bacterium regulates various metabolic pathways, particularly those related to glucose and proline metabolism, and supports oxidative phosphorylation. In the absence of the bacterium, there is an increase in glycolytic flux, resulting in the activation of overflow metabolism (Fig. 6). This study reveals new aspects of the metabolic coevolution of *A. deanei* with its symbiont and provides evidence about the origin of cellular structures in eukaryotic cells. Furthermore, comparisons of monoxenous and heteroxenous metabolism have raised important questions about the evolution of parasitism in the Trypanosomatidae family.

## HIGHLIGHTS

- This study illustrates how the bacterial symbiont impacts the energy metabolism of *A. deanei*.
- The symbiont benefits from the metabolic intermediates of glycolysis and optimizes the host oxidative metabolism by regulating the flow of pyruvate to the mitochondrion.
- The aposymbiotic strain depends on fermentative metabolism for the synthesis of energy molecules.
- In the absence of the bacterium there is an increase in glycolytic flux, resulting in the activation of overflow metabolism.
- The symbiotic bacterium contributes to the metabolic integration of mitochondrion and glycosomes.
- The endosymbiosis in trypanosomatids gives insight into the symbiotic origin of organelles.
- Comparison of the energy metabolism of monoxenic and heteroxenic trypanosomatids reveals new clues about the evolution of parasitism.

## SUPPLEMENTAL MATERIAL

Supplemental figures msphere.00457-25-s0001.doc Figures S1-S3.

Supplemental legends msphere.00457-25-s0002.docx Legends to supplemental figures and table.

Table S1 msphere.00457-25-s0003.xlsx Proteomic data.

ASM does not own the copyrights to Supplemental Material that may be linked to, or accessed through, an article. The authors have granted ASM a non-exclusive, world-wide license to publish the Supplemental Material files. Please contact the corresponding author directly for reuse.
