# Supplementary figures and images for: Endosymbiosis in trypanosomatids: the bacterium regulates the intermediate and oxidative metabolism of the host cell
Source: mSphere. 2025 Oct 13;10(11):e00457-25. doi: 10.1128/msphere.00457-25 (PMC12645908; doi:10.1128/msphere.00457-25)

**Supplementary Figures**

**­**


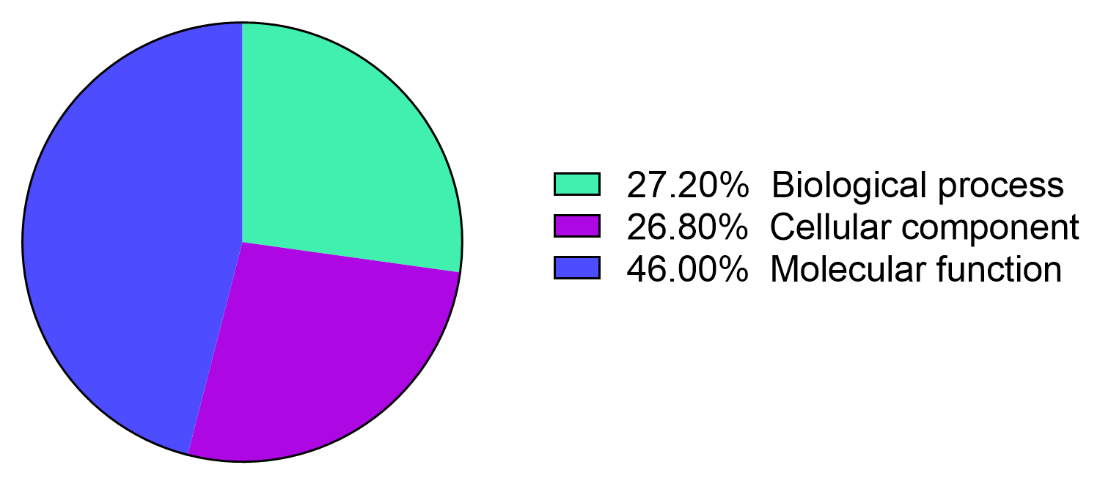
**Figure S1**

**
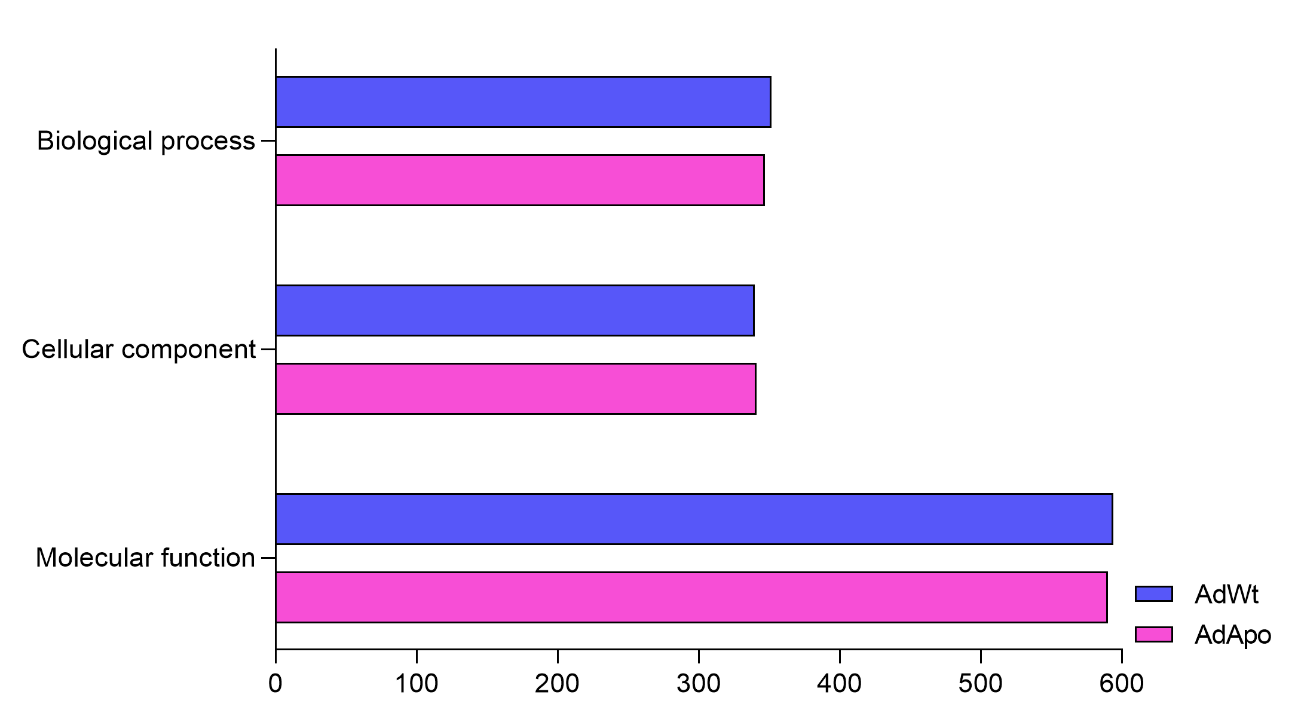
**

**Figure S2**


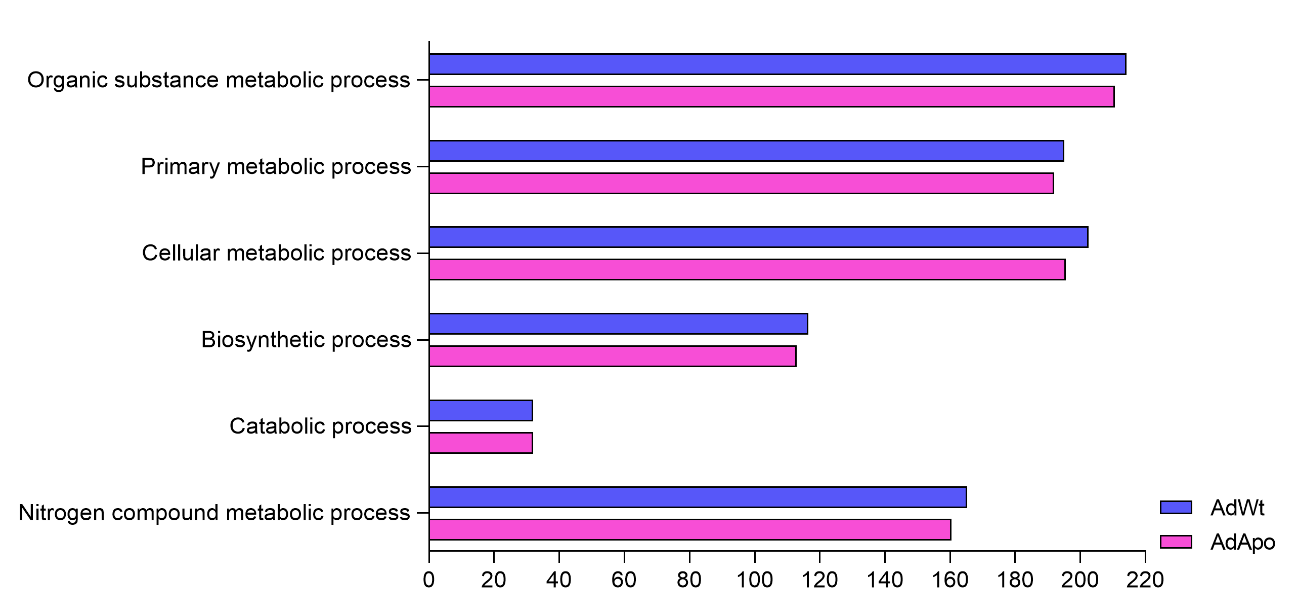
**Figure S3**

Supplement: Supplemental figures — Figures S1-S3. [file msphere.00457-25-s0001.doc]
