# Supplementary material for: Endosymbiosis in trypanosomatids: the bacterium regulates the intermediate and oxidative metabolism of the host cell
Source: mSphere. 2025 Oct 13;10(11):e00457-25. doi: 10.1128/msphere.00457-25 (PMC12645908; doi:10.1128/msphere.00457-25)
Supplement: Supplemental legends — Legends to supplemental figures and table. [file msphere.00457-25-s0002.docx]

**Supplemental legends to figures and table**

Figure S1: Main categories of proteins. GO analysis divided the proteome into 3 main categories: 46% of proteins with molecular function (blue), 27.2% participating in biological processes (cyan) and 26.8% in cellular components (purple).

Figure S2: Predicted functions for *p*roteins. GO analysis *A. deanei* proteins of the wild-type (blue bar) and aposymbiotic (pink bar) strains.

Figure S3: Predicted metabolic processes. Metabolic processes were subdivided as organic substances, primary, cellular, biosynthetic, catabolic or nitrogenous compounds. Wild-type cells (blue bar) and aposymbiotic strains (pink bar).

Table S1: Proteomic data comparing AdWt and AdApo strains with reference to Vulcano plot. Pink sheet: proteins with p value less than 0.05; purple sheet: low abundant proteins; blue sheet: proteins more abundant in one strain than the other, with low statistical power; cyan sheet: proteins equally abundant in both *A. deanei* strains.
